# Comparative Analysis of the Occurrence of Depression, Stress, and Anxiety in Pregnant Women Requiring Hospitalization and Those Not Hospitalized

**DOI:** 10.3390/jcm14217865

**Published:** 2025-11-05

**Authors:** Agnieszka Ptak, Kinga Przylibska, Małgorzata Stefańska, Joanna Kowalska

**Affiliations:** 1 Faculty of Physiotherapy, Wroclaw University of Health and Sport Science, 51-612 Wroclaw, Poland; kinga.przylibska@gmail.com (K.P.); malgorzata.stefanska@awf.wroc.pl (M.S.); joanna.kowalska@awf.wroc.pl (J.K.); 2 Falkiewicz Specialist Hospital, 52-114 Wroclaw, Poland

**Keywords:** depression, stress, anxiety, labor anxiety, pregnant women, fatigue

## Abstract

**Background/Objectives:** The aim of the study was to assess the emotional state (stress, mood, and anxiety level, including labor anxiety) of pregnant women depending on the course of pregnancy and the related place of stay (hospital pregnancy pathology department, home). **Methods:** A total of 100 participants were recruited between 25 and 38 weeks of pregnancy. A total of 88 fully completed questionnaires of women qualified for analysis, including 45 women staying in the hospital (G1) and 43 women who did not require hospitalization (G2). The Depression Anxiety Stress Scale (DASS-42), the Labor Anxiety Questionnaire (KLP II), the Fatigue Assessment Scale (FAS), and a self-administered questionnaire were used. **Results:** All subjects showed an average moderate level of depression and stress and a high level of anxiety. A statistically significant difference in mood level (DASS depression) was noted between group G1 and group G2 (*p* = 0.0217). About 35% of all subjects in total and both groups achieved a result indicating a severe or extremely severe level of stress. About 66% of subjects in both groups showed a severe and extremely severe level of anxiety. None of the women studied had values interpreted as a physiological level of anxiety. **Conclusions:** Regardless of the course of pregnancy and the related place of residence, the risk of emotional disorders is high. It seems reasonable to perform screening tests on pregnant women to identify those who may or already have these problems.

## 1. Introduction

Pregnancy is a time of intense physical, mental, hormonal, and social changes that can affect mood swings and the occurrence of mental disorders.

Pregnancy is a time of changes and new challenges for the future mother and is socially perceived as a joyful period of waiting. Psychological well-being studies conducted on groups of pregnant women indicate that for some women it is a time of joyful waiting and self-fulfillment, but for others, it is a source of emotional difficulties and mental problems. Among the latter group, there is a significant percentage of respondents who have been diagnosed with symptoms of depression and/or anxiety during pregnancy [1,2]. Women’s mental health and emotional well-being have an impact on the course of pregnancy, childbirth, the condition of the newborn, the initiation and successful continuation of breastfeeding, as well as the further development of the relationship between mother and child [3]. Women diagnosed with emotional disorders such as depression and anxiety have a greater tendency for cesarean section, a higher level of fear of childbirth, eating disorders, difficulties in developing strategies for coping with difficult situations, and risk of suicide [4,5,6,7,8].

The increased risk of depression and anxiety in pregnant women is more common in women who have had depression earlier in life [3]. In addition, untreated depression and anxiety during pregnancy can lead to fetal heart rhythm disturbances, fetal hyperactivity, premature birth, impaired neonatal development, reduced neonatal activity after birth, failure to start breastfeeding, or not continuing breastfeeding after 6 weeks of life [9,10,11]. At the same time, depression or anxiety can lead to intrauterine growth retardation, premature birth, and low birth weight [5,8]. The occurrence of symptoms of depression as well as high levels of anxiety may cause placental dysfunction and affect brain development and poor cognitive development of offspring [3,8,12,13]. In addition, anxiety and depression diagnosed during pregnancy predispose mothers to the development of severe postpartum psychosis, one of the most severe disorders diagnosed in the postpartum period [7,14,15]. Also, taking antidepressants during pregnancy may pose a risk of autism in children as well as depressive and anxiety disorders in teenagers [3,16,17,18]. Studies conducted on various social groups also point to the higher incidence of depressive and anxiety disorders in low-income countries, in women who are not in relationships, in women whose pregnancy was unplanned, and in those with lower education [19,20].

The variety of factors reported by researchers that are related to increased stress levels, the development of depressive disorders and anxiety disorders in pregnant women, and the lack of clear results of previous studies encourage their continuation, taking into account not only generalized anxiety but also labor anxiety. Labor anxiety is a specific psychological dimension different from general anxiety and, according to some researchers, may intensify or be a symptom of perinatal depression [21,22].

Early recognition and diagnosis of emotional disorders allow for the implementation of rapid preventive and/or therapeutic measures [1,20] and planning professional obstetric care. Therefore, this study aimed to assess the emotional state (stress, mood, and anxiety level, including labor anxiety) of pregnant women depending on the course of pregnancy (physiological, with problems) and the related place of stay (hospital pregnancy pathology department, home) and to identify factors related to their mood and anxiety level. The results of the study may identify pregnant women who require additional interventions and support in the emotional area and factors potentially related to them. This will allow for the rapid initiation of psychoprophylactic measures and, as a consequence, may significantly improve the quality of labor and the condition of the newborn.

## 2. Material and Methods

### 2.1. Study Group

The study was conducted at the A. Falkiewicz Specialist Hospital, ul. Warszawska 2 in Wrocław, between January–December 2024.

The study involved 100 women who met the following inclusion criteria: informed consent to participate in the study, being at the age of ≥18 years, women between 25 and 39 weeks of pregnancy, consent of the attending physician to participate in the study (applies to women staying in the hospital in the pregnancy pathology ward), and no communication difficulties. Exclusion criteria were a mental deficiency, having a diagnosis of psychiatric disease, especially depression or/and anxiety disorders, and their treatment, and incomplete study questionnaires. None of the study participants were taking antidepressants or anxiolytic medications. No woman’s condition was life-threatening to her or her baby, and none of the patients’ conditions were classified as urgent.

The women were informed about the assumptions and goals of the study and gave their voluntary, written consent to participate in it. The study was conducted according to the guidelines of the Declaration of Helsinki and received approval from the Senate Commission for the Ethics of Scientific Research of the University Health and Sport Sciences in Wroclaw 24/2025.

Finally, 88 fully completed questionnaires of women were qualified for analysis, including 45 women staying in the pregnancy pathology ward (G1) and 43 women who did not require hospitalization but reported for check-ups to a gynecologist, constituting a comparison group (G2). Both groups did not differ from each other in general, except for place of residence and participation in childbirth classes (Table 1).

### 2.2. Measurement Tools

The Depression Anxiety Stress Scale (DASS-42), the Labor Anxiety Questionnaire (KLP II), the Fatigue Assessment Scale (FAS), and a self-administered questionnaire were used. The self-administered questionnaire included basic socio-demographic data (i.e., age, education, marital status, type of employment) and questions related to pregnancy, attending childbirth classes, and physical activity.

DASS-42 in the Polish adaptation by Makary-Strudzińska et al., [23] used to assess symptoms of depressive–anxiety disorders, consists of 42 tasks divided into 3 symptom groups (depression scale, anxiety scale, stress scale) with 14 affirmative tasks each. The examined person responds to the tasks taking into account the last week preceding the study using a 4-point Likert scale (where 0 means “it did not apply to me at all”, and 3 means “it applied to me to a very large extent or most of the time”). The result is calculated separately for each scale and ranges from 0 to 42 points. A higher result is an indicator of a greater intensity of the examined feature. The overall result of the study is the sum of points from 3 scales and ranges from 0 to 126 points, converted to a sten scale. Very low scores are 1 sten, low scores are 2–3 sten, reduced scores are 4 sten, average scores are 5–6 sten, increased scores are 7 sten, high scores are 8–9 sten, and very high scores are 10 sten. In the non-clinical sample, Cronbach’s alpha coefficient was 0.96 for the DASS-42 total score [23].

KLP II asses the level of labor anxiety in pregnant women and consists of 9 items, The examined person selects one of four response categories (definitely yes, rather yes, rather no, definitely no) referring to current feelings. The final score ranges from 0 to 27. A higher score indicates a higher intensity of labor anxiety. A score of up to 13 points indicates a low level of labor anxiety, a score of 14–15 points indicates a slightly elevated level of anxiety, a score of 16–17 points indicates a high level, and scores above 18 points indicate a very high level of labor anxiety. Cronbach’s alpha reliability coefficient is satisfactory and amounts to 0.69 [22].

FAS (Fatigue Assessment Scale) consists of 10 items and is used to assess fatigue. The subject indicates how they felt by selecting the appropriate answer on a 5-point Likert scale (where 1 point means “never” and 5 points “always”). The final score is between 10 and 50 points. The higher the score, the greater the fatigue. A score of 22–34 points indicates mild/moderate fatigue, and a score above 34 points indicates severe fatigue and tiredness. Cronbach’s α coefficient for the original FAS scale is 0.87, and in the studies using the Polish translation, Cronbach’s α was 0.86 [24,25].

### 2.3. Statistical Analysis

The analysis of quantitative data was preceded by checking the normality of the distribution of variables. Based on the result of the Shapiro–Wilk test, the mean was used as a measure of central tendency, the standard deviation (SD) was used as a measure of dispersion, and the significance of differences between groups was estimated using the *T*-test for independent samples. For all comparisons, the Leaven test confirmed the homogeneity of variance.

The median was used as a measure of central tendency for ordinal variables, and the interquartile range (IQR = Q3 − Q1) was used as a measure of dispersion. The significance of differences between groups for this type of variable was checked using the Mann–Whitney U test.

To describe nominal variables, the numbers of individual categories and percentage values were presented. Differences between groups were checked using the Chi-square test. The influence of selected factors on the level of mood and anxiety (DASS depression, DASS anxiety) was identified using multivariate regression models.

Effect size for the Mann–Whitney U test was calculated using Cohen’s d coefficient, and for the Chi-square test, Cramer’s V coefficient was used. All analyses were performed in the Statistica program (14.1.0.4) and PQ-Stat 1.8.4. Effect size was calculated using statistical calculators online: https://www.statskingdom.com/effect-size-calculator.html (accessed on 23 October 2025) and https://www.psychometrica.de/effect_size (access on 23 October 2025). The significance level for all analyses was set at *p* < 0.05.

## 3. Results

The analysis of the DASS-42 scale results showed an average moderate level of depression and stress (7 sten) and a high level of anxiety (8 sten) in all the subjects. The total DASS-42 score for all the subjects showed a moderate level. Group G1 differed significantly from group G2 in a higher score on the depression scale (DASS depression). However, both results were within the range of 7 sten (Table 2).

The qualitative analysis indicated a greater differentiation in results between the groups. The qualitative analysis of the depression scale (DASS depression) confirmed a moderate (5–6 sten) level of depression in all subjects and in both groups. Additionally, it was shown that almost 30% of all subjects achieved a result indicating serious and very serious depression. In 40% of the subjects from group G1, severe symptoms of depression (8–9 sten) were observed, while severe and extremely severe symptoms of depression (≥8 sten) were observed in almost 20% of the subjects from G2 (Table 3).

The qualitative analysis of the anxiety scale (DASS anxiety) showed, both in all subjects together and in both groups, severe and extremely severe levels of anxiety in about 66% of the subjects. None of the subjects recorded values equal to or lower than 4 sten, interpreted as a physiological level of anxiety (Table 3).

The level of stress (DASS stress) in most of the subjects was low or moderate. About 35% of all subjects in total and both groups achieved a result indicating severe or extremely severe levels of stress (Table 3). Analysis of the total score of DASS (DASS total) showed severe and extremely severe symptoms (8–10 sten) in over 50% of the subjects in total and in both groups. A low result (≤4 sten) was observed only in almost 15% of all subjects. However, it was more than half as common in group G1 compared to group G2 (9% vs. 21%) (Table 3).

Qualitative analysis of the level of experienced labor anxiety assessed with the KLP scale showed low or slightly elevated values in over 80% of all subjects in total and in both groups. High and very high values were recorded in about 20% of subjects from group G1 and 11% from group G2 (Table 4).

The tests showed mild fatigue in over 50% and severe fatigue in almost 10% of women in both groups. The differences between the groups were not statistically significant (Table 5).

The performed regression analysis and the created multivariate regression models did not show a significant influence of any factor (among them were age, gestational week, number of pregnancies, physical activity, labor anxiety, fatigue, as well as marital status, education, dwelling place, and work) on the level of anxiety and depression (DASS depression and DASS anxiety).

## 4. Discussion

Pregnancy is a state in which there is an increased risk of depression and anxiety disorders as well as frequent mood changes. Complications during pregnancy and the need for hospitalization are stress factors that can intensify the intensity of negative emotions in pregnant women [26,27]. The presented research results partially confirm this thesis. Significantly worse mood was noted in hospitalized women compared to the group of women staying at home. This was also confirmed by qualitative analysis, which showed significantly more cases of depression symptoms among women staying in the ward compared to the non-hospitalized group (40% vs. 20%). Similar results regarding the level of depression were also obtained in their studies by Dennis (2017) and Redinger (2020) [28,29].

These symptoms were accompanied by high and very high levels of anxiety, occurring in as many as approx. 66% of the women studied. Interestingly, the level of anxiety in the group of non-hospitalized women was equally high and also reached approx. 66%. These results are partially consistent with the results of other authors because many of them noted a smaller percentage of women with low mood and high anxiety but with a definite predominance of anxiety disorders. Thus, in the studies of Testouri et al. [26], the prevalence of depressive symptoms was 20%, and that of anxiety symptoms was 39%. Nagandla et al. indicate that the most common mental health problems are anxiety at 18.8% depression at 6.9%, and stress at 4.2% [29]. Lewicka et al. noted almost 22% of women with symptoms of depression and over 48% had an increased level of anxiety [26,27,30]. In contrast, Toscano et al. in their meta-analysis indicate that the prevalence of depression was 34%, and that of anxiety was 29% [30]. Additionally, the authors suggest that one in three women hospitalized during pregnancy due to obstetric complications report symptoms of depression or anxiety, which is twice the reported prevalence of antenatal depression in the general obstetric population. This confirms the present report [31,32].

However, the DASS scale total score showed high and very high results (approx. 50%) in both study groups, similar to the above-mentioned percentage level of anxiety. Further comparative analysis showed that low DASS scores were recorded twice as often in the group of hospitalized women, while it should be noted that none of the study participants had values interpreted as a physiological level of anxiety (equal to or lower than 4 sten). The stress level was also comparable in both groups, reaching a heightened level of stress. The percentage of cases of women with severe and extremely severe stress levels was very similar and statistically insignificant (35.6% vs. 37.3%), although it is worth noting that cases of extreme stress levels were recorded only in the group of non-hospitalized women (4.7%). Similar results of high stress levels were shown by Postępska et al. (32.1%) and Awad-Sirhan et al. (32.6%) [24,30,31,32].

Therefore, a hospital stay does not always have to be a stress factor. Lewicka et al. also found that the obstetric situation does not determine the intensity of negative emotions among patients with high-risk pregnancies [25,31].

Some studies show that hospitalization can reduce anxiety and depressive symptoms in pregnant women because their health problems are addressed and monitored by medical staff, especially when their health condition stabilizes over time [32].

In this study, no significant differences were noted in the level of fatigue, which was at a mild level, as well as in labor anxiety. However, it should be noted that a higher percentage of cases of high labor anxiety was noted in the group staying in the hospital (20% vs. 11%). In the entire group of women studied, the level of anxiety was low or slightly elevated, similar to the studies by Gebuza et al. [33] and the studies by Kaźmierczak et al. [19,21,33].

Among the analyzed factors (such as age, week of pregnancy, number of pregnancies, physical activity, labor anxiety, and fatigue), it was not possible to identify those that significantly affect the level of anxiety and mood in the entire study group of women and in both subgroups of hospitalized and non-hospitalized women. It seems that pregnancy itself and the approaching delivery are a sufficiently stressful period in a woman’s life, which can intensify negative emotions in the form of low mood or high anxiety, even when its course is physiological. Pregnancy is in 12th place out of 43 listed on the Social Readjustment Scale, which causes the highest level of stress in the respondents [18]. This may be the reason for the lack of such clear differences in the results of both groups compared and the difficulties in identifying the most important factors. This was probably also related to the small group of respondents.

Nevertheless, it is worth continuing the above studies, because emotional problems and mental disorders are still often trivialized and not diagnosed, and the symptoms of illness are explained by fatigue and/or hormonal disorders characteristic of pregnancy [13,34,35,36].

Screening tests and interventions dedicated to pregnant women regardless of the course of pregnancy and place of stay (hospital, home) are important to identify and alleviate even the smallest negative emotional effects.

### Limitations

The presented studies have certain limitations. The scales used in the study were of a screening nature and did not bear the hallmarks of a medical diagnosis. Only selected factors that may be related to the emotional state of the pregnant women studied were taken into account. Due to the size of the sample, the reasons for hospitalization were not taken into account, which should be taken into account in subsequent studies, as well as increasing the number of women studied. The study was a one-off and does not show the dynamics of changes in the emotional state of pregnant women, which should also be taken into account in future studies.

## 5. Conclusions

In the study group of pregnant women staying in the hospital, significantly worse mood and significantly more cases of depression symptoms were noted compared to women who were not hospitalized.

A high level of anxiety was shown in approx. 66% of cases in both study groups.

It seems reasonable to perform screening tests on pregnant women, regardless of their medical condition and place of stay, to identify those who are at risk of developing emotional disorders. It is recommended to implement routine emotional health screenings during prenatal visits, ensure the systematic integration of psychological support within obstetric wards, and develop comprehensive psychoeducation programs for expectant mothers.

## Figures and Tables

**Table 1:** Characteristics of the study group.

		All Group n = 88		Group G1 n = 45		Group G2 n = 43		G1 vs. G2	
		Mean	SD	Mean	SD	Mean	SD	T	*p*
age (years)		31.36	3.97	30.89	3.97	31.86	3.96	−1.15	0.254
week of pregnancy/gestation period		32.63	3.70	33.36	3.72	31.86	3.56	1.92	0.058
stay in the ward (days)		5.64	7.34	5.64	7.34	-	-		
		n	%	n	%	n	%	χ^2^	*p*
marital status	in a relationship	85	96.6	45	100.00	40	93.0	5.69	0.1274
	single	3	3.4	0	0.00	3	7.0		
education	primary	1	1.1	1	2.2	0	0.0	11.81	0.008 *
	vocational	2	2.3	0	0.00	2	4.7		
	secondary	10	11.4	9	20.0	1	2.3		
	higher	75	85.2	35	77.8	40	93.0		
Dwelling place	village	21	23.9	15	33.3	6	14.0	8.84	0.012 *
	city < 100 k	6	6.8	5	11.1	1	2.3		
	city > 100 k	61	69.3	25	55.6	36	83.7		
work	unemployed	3	3.4	3	6.7	0	0.0	9.95	0.268
	working on sick leave	80	90.9	40	88.9	40	93.0		
	working	5	5.7	2	4.4	3	7.0		
which pregnancy	1	66	75.0	28	62.2	38	88.4	10.82	0.055
	2	16	18.2	12	26.7	4	9.3		
	3 and >3	6	6.8	5	11.1	1	2.3		
course of pregnancy	physiological	72	81.8	29	64.44	43	100.00	18.69	<0.001 *
	at risk	16	18.2	16	35.56	0	0.00		
fertilization	natural	83	94.3	42	93.3	41	95.3	9.65	0.086
	supported	5	5.7	3	6.7	2	4.7		
participation in a childbirth class	yes	62	70.5	19	42.2	43	100.0	35.26	<0.001 *
	no	26	29.5	26	57.8	0	0.0		
physical activity before pregnancy	yes	75	85.2	36	80.0	39	90.7	2.05	0.152
	no	13	14.8	9	20.0	4	9.3		

G1—women staying in the pregnancy pathology ward, G2—pregnant women who did not require hospitalization, * *p* < 0.05.

**Table 2:** Quantitative analysis of the level of depression, anxiety, stress, and fatigue estimated by the DASS scale.

Scales:	All n = 88			G1 n = 45			G2 n = 43			G1 vs. G2		Effect Size
	Median	IQR	sten	Median	IQR	sten	Median	IQR	sten	U	*p*	Cohen’s d
DASS depression	17	4	7	18	4	7	16	4	7	692	0.022 *	**0.50**
DASS anxiety	20	6.5	8	20	6	8	20	6	8	877	0.453	0.16
DASS stress	21.5	9	7	22	9	7	21	9	7	934.5	0.786	0.06
DASS-42 total	59	17	8	60	18	8	59	18	8	846	0.313	0.22
KLP total	12	4	-	12	4	-	12	5	-	889	0.515	0.14
FAS total	23	7.5	-	24	9	-	23	6	-	961	0.960	0.01

G1—women staying in the pregnancy pathology ward, G2—pregnant women who did not require hospitalization, DASS-42—the Depression Anxiety Stress Scale, KLP II—the Labor Anxiety Questionnaire, FAS—the Fatigue Assessment Scale, * *p* < 0.05.

**Table 3:** Qualitative analysis of DASS depression.

DASS Depression	All		G1		G2		G1 vs. G2		Effect Size
	n	%	n	%	n	%	Chi^2^	*p*	Cramer’s V
low	17	19.3	6	13.3	11	25.6	14.54	0.002 *	0.41
moderate	45	51.2	21	46.7	24	55.8			
severe	22	25.0	18	40	4	9.3			
extremely severe	4	4.5	0	0	4	9.3			
**DASS anxiety**									
normal	0	0	0	0	0	0	0.07	0.968	0.03
moderate	29	32.9	15	33.3	14	32.6			
severe	46	52.3	23	51.1	23	53.5			
extremely severe	13	14.8	7	15.5	6	13.9			
**DASS stress**									
low	40	45.5	19	42.2	21	48.8	3.19	0.363	0.19
moderate	16	18.2	10	22.2	6	13.9			
severe	30	34.1	16	35.6	14	32.6			
extremely severe	2	2.3	0	0	2	4.7			
**DASS total**									
low	13	14.8	4	8.9	9	20.9	6.27	0.100	0.27
moderate	28	31.8	16	35.6	12	27.9			
severe	44	50.0	25	55.6	19	44.2			
extremely severe	3	3.4	0	0	3	7.0			

G1—women staying in the pregnancy pathology ward, G2—pregnant women who did not require hospitalization, DASS-42—the Depression Anxiety Stress Scale, * *p* < 0.05.

**Table 4:** Qualitative analysis of the level of postpartum anxiety assessed using the KLP scale.

KPL Total	All		G1		G2		G1 vs. G2		Effect Size
	n	%	n	%	n	%	Chi^2^	*p*	Cramer’s V
low	56	63.6	29	64.4	27	62.8	2.07	0.559	0.15
slightly double	18	20.5	7	15.6	11	25.6			
high	11	12.5	7	15.6	4	9.3			
very high	3	3.4	2	4.4	1	2.3			

G1—women staying in the pregnancy pathology ward, G2—pregnant women who did not require hospitalization, KLP II—the Labor Anxiety Questionnaire.

**Table 5:** Qualitative analysis of the fatigue level estimated by the FAS scale.

FAS Total	All		G1		G2		G1 vs. G2		Effect Size
	n	%	n	%	n	%	Chi^2^	*p*	Cramer’s V
lacking	36	40.9	18	40.0	18	41.9	0.05	0.978	0.15
mild	44	50.0	23	51.1	21	48.8			
severe	8	9.1	4	8.9	4	9.3			

G1—women staying in the pregnancy pathology ward, G2—pregnant women who did not require hospitalization, FAS—the Fatigue Assessment Scale.

## Data Availability

The data that support the findings of this study are not openly available due to reasons of sensitivity and are available from the corresponding author upon reasonable request. Data are located in controlled-access data storage.
